# The KEAP1/NRF2 Signaling Pathway in Keratinization

**DOI:** 10.3390/antiox9080751

**Published:** 2020-08-14

**Authors:** Yosuke Ishitsuka, Tatsuya Ogawa, Dennis Roop

**Affiliations:** 1 Department of Dermatology, Faculty of Medicine, University of Tsukuba 1-1-1 Tennodai, Tsukuba, Ibaraki 305-8575, Japan; tatsuya.ogawa.1220@gmail.com; 2 Department of Dermatology and Charles C. Gates Center for Regenerative Medicine, University of Colorado Anschutz Medical Campus, Aurora, CO 80045, USA; dennis.roop@cuanschutz.edu

**Keywords:** gene expression regulation, environmental response, squamous epithelium, KEAP1/NRF2 signaling, keratinization, thiol, disulfide, loricrin

## Abstract

Keratinization is a tissue adaptation, but aberrant keratinization is associated with skin disorders such as ichthyoses, atopic dermatitis, psoriasis, and acne. The disease phenotype stems from the interaction between genes and the environment; therefore, an understanding of the adaptation machinery may lead to a new appreciation of pathomechanisms. The KEAP1/NRF2 signaling pathway mediates the environmental responses of squamous epithelial tissue. The unpredicted outcome of the *Keap1*-null mutation in mice allowed us to revisit the basic principle of the biological process of keratinization: sulfur metabolism establishes unparalleled cytoprotection in the body wall of terrestrial mammals. We summarize the recent understanding of the KEAP1/NRF2 signaling pathway, which is a thiol-based sensor-effector apparatus, with particular focuses on epidermal differentiation in the context of the gene-environment interaction, the structure/function principles involved in KEAP1/NRF2 signaling, lessons from mouse models, and their pathological implications. This synthesis may provide insights into keratinization, which provides physical insulation and constitutes an essential innate integumentary defense system.

## 1. Introduction and Overview

### Keratinization as an Environmental Response: Beneficial or Detrimental

“The stratum corneum (SC) is a magnificent example of the successful adaptation of a tissue. Its efficient function is a prerequisite for life itself. We depend on its control of the movement of water through the skin and on its protective role in the prevention of penetration by pathogens or harmful substances. Apart from its functional importance, there is no escaping the fact that the most common skin disorders (psoriasis, eczema, inherited disorders of keratinization, acne) are characterized by abnormal keratinization and/or scaliness.” This quotation appears in the preface of the book titled “Stratum Corneum,” which was initially published in 1982 [1]. The SC functions as a specialized insulation barrier that gives rise to unparalleled mechanical resilience and impermeability [2]. The functional and structural analogy is the “bricks and mortar” model; bricks correspond to corneocytes (terminally differentiated keratinocytes), and mortar represents lipid bilayers provided from the lamellar granule secretory system located in the uppermost living layer, the stratum granulosum (SG) [2]. Over the last four decades, medical genetics has identified a range of predisposing factors that shape disease phenotypes. The prime example would be autosomal recessive congenital ichthyosis (ARCI), which is caused by inborn errors in the formation/function of the SC lipid permeability barrier [3]. The genotype-phenotype correlation in ARCI unequivocally suggested that the functional “mortar” is mandatory for desiccation tolerance. Although the degrees of severity may vary among afflicted individuals, the universal phenotype is hyperkeratosis; however, it should be noted that dry, scaly skin is a consequence of biological responses to a breach of the SC permeability barrier, rather than a functional deficit [4,5,6]. Similarly, autosomal dominant ichthyosis vulgaris (IV; common dry skin) requires factors other than filaggrin (Flg)-null variants [7] to manifest as atopic dermatitis (AD; eczema) or other allergic conditions [8]. Therefore, as Marks and Plewig noted [1], keratinization and its disorders encompass essential environmental responses, the product of a gene-environment interaction [9].

Because the epidermis comprises a frontline defense system and interacts with the external environment, it is not surprising that xenobiotic keratinocyte responses affect keratinization, which involves both phase I [10] and phase II metabolism [11]. However, the profound relationship between keratinization and the Kelch-like erythroid cell-derived protein with the cap ’n’ collar homology-associated protein 1 (KEAP1)/NFE2-related factor 2 (NRF2) signaling pathway, demonstrated in KEAP1-deficient mice [11], was unexpected at the beginning of the twenty-first century, even for keratinocyte biologists [12].

The KEAP1/NRF2 signaling pathway, a thiol-based sensor-effector apparatus [13], promptly responds to the redox environment and acclimates the organism to the ever-changing external environment. Although redox-based regulation of gene expression maintains tissue homeostasis by promoting epidermal barrier repair [14,15], it is becoming increasingly apparent that aberrant activation of NRF2 signaling could be detrimental and could lead to a plethora of skin diseases [11,16,17,18]. This review aims to discuss the KEAP1/NRF2 signaling pathway’s function with a particular focus on epidermal homeostasis and disease as a successful adaptation [1,2] or a gene-environment mismatch [9], respectively.

## 2. Keratinization at a Glance

### Epidermal Differentiation; a Search for the Thiol-Rich Protein

Since the early twentieth century, it has been commonly understood that epidermal differentiation involves sulfur metabolism; thiol (-SH) groups of the proliferative layer are converted to covalent disulfide (-S-S-) bridges of the keratin molecule [19]. This simple concept remains central to our understanding of the most critical epidermal terminal differentiation process, keratinization. Although it had long been known that keratinized materials, such as the callus, are markedly insoluble against alkaline cleavage of disulfides [20], the other crucial biochemical property of keratinization remained an enigma. In the 1970s, it was found that membrane-like insoluble materials, i.e., cornified cell envelopes (CEs), are a mixture of proteins bound together via ε-(γ-glutamyl) lysine cross-linkages [21]. Later, using keratinocytes cultured under submerged conditions, involucrin (IVL) was identified as a soluble cell envelope precursor [22]. However, “true” keratinization is promoted by the ambient air that oxidizes thiols [20], and a thiol-rich major CE precursor was hypothesized [19,23,24,25,26]; this was confirmed with its actual discovery in 1990 when Mehrel and Roop et al. cloned and characterized the long-sought sulfur-rich CE precursor loricrin (LOR) [27].

**Figure 1:**
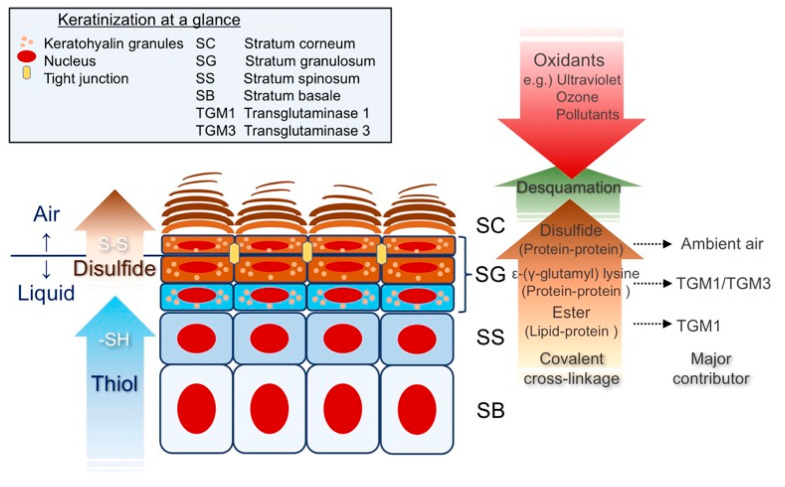
Keratinization at a glance. A thiol (-SH) gradient through the epidermis provides unparalleled cytoprotection. Following a microenvironmental rise in Ca^2+^, TGMs catalyze ε-(γ-glutamyl) lysine (protein-protein) cross-linkages and ester (lipid-protein) cross-linkages above the SS. Upon exposure to the ambient air, the keratinocyte cytoskeleton undergoes extensive disulfide formation in the SG and stabilizes corneocytes.

In the epidermis, keratinocytes that exit from the proliferative basal layer migrate upward through the SG, which is the last living layer [28]. Before being completely enucleated and flattened [29], long-chain ω-hydroxyceramides are covalently attached to CE scaffolds, such as IVL, on the corneocyte cell surface, via ester cross-linkage [2,30]. Subsequently, cytoskeletal proteins are further condensed via ε-(γ-glutamyl) lysine [20] and disulfide cross-linkages [19], leading to a CE formation that is observed as electron-dense deposits that replace the keratinocyte cell membrane. This critical differentiation process mostly occurs in the SG and allows keratinocytes to withstand the harsh, arid external environment [2] (Figure 1). This differentiation process may be analogous to vertebrate evolution, given that terrestrial hominins are considered to have emerged from aquatic ancestors [31]. Along the same line of reasoning, a recent piece of phylogenic evidence suggests that only the amniotes (birds, reptiles, and mammals), but not fish or amphibians, have the gene cluster known as the epidermal differentiation complex (EDC) [32]. The SG is rich in keratohyalin granules that come in two flavors: the histidine-rich F-granule and the sulfur-rich L-granule [33]. Keratohyalin granules thus harbor distinct gene products derived from the epidermal differentiation complex [33]; the F-granule contains a cationic protein that aggregates the keratin intermediate filament, filaggrin (FLG) [34], while the L-granule contains a sulfur-rich protein LOR [27]. The insolubility of corneocytes is primarily attributable to the ε-(γ-glutamyl) lysine cross-linkage formed between LOR and other prospective cross-linkage partners, keratins K1/K10 or FLG [35,36]. Thus, LOR organizes the higher-order structure of corneocytes [37]. Upon exposure to ambient air, LOR stabilizes corneocytes via extensive disulfide (-S-S-) cross-linkage formation, and possibly auto-oxidation [27,37], thus completing epidermal differentiation.

## 3. The KEAP1/NRF2 Signaling Pathway and the EDC

Gene-environment interaction is a core concept of Darwinian evolution and can be applied universally to a myriad of biological phenomena [38]. Every gene strategically undergoes adaptive changes, such as duplication or deletion, in response to selective pressure from a given environment and results in functional specialization and diversification [39]. However, because generations of recombination events are required to ensure efficient passage of germline mutations to progeny on a large enough scale, we are also equipped with another, rapid mode of adaptive responses: regulation of gene expression.

### 3.1. The Evolution of the EDC Gene Cluster; Analogy to the β-globin Gene Cluster

The “β-like-globin” genes (hemoglobin β-chain gene cluster) are a classic example of gene-environment interaction. Living organisms have acquired multitiered adaptive strategies and adapted to variable degrees of oxygen demand, such as development/growth [40], hypoxic environments [41], or endemic malaria [42]. The recognition and characterization of the locus control region for transcriptional regulation accelerated the investigation of the *cis*-acting element located in the 5′ region of the β-globin gene cluster, leading to the identification of transcription factors (TFs) such as GATA-binding protein 1 (GATA1), nuclear factor erythroid 2 (NFE2), and NFE2-related factor 2 (NRF2) [43,44]. It was assumed that basic leucine zipper (bZIP) TFs specifically regulate erythropoiesis; however, in contrast to germline deletion of *Gata1* [45] or *Nfe2* [46], *Nrf2*-deficient mice exhibited normal erythropoiesis [47]. Rather, the tissue distribution of NRF2 is ubiquitous, and the NRF2-binding sequence resembles antioxidant response elements (AREs) [13], which are the *cis*-acting elements for phase II detoxifying enzyme expression. This reasoning by Ito et al. led to the conclusion that NRF2 is a general regulator of phase II detoxification [48] and the characterization of the KEAP1/NRF2 signaling pathway. Cysteine residues of KEAP1 act as sensors for electrophilic assault and NRF2 is the effector [13,49]. The thiol-rich protein KEAP1 subjects NRF2 to ubiquitin-mediated proteasomal degradation in the cytosol and represses NRF2 in steady-state [13]. The nucleophilic KEAP1 residues sense various electrophilic assaults, and then NRF2 coordinately induces a battery of cytoprotective genes [13].

Epithelial tissues constantly undergo detoxification [48]. Indeed, NRF2-deficient mice are susceptible to chemical carcinogens [50,51,52] that activate phase II detoxification. However, the consequence of the loss of the cytosolic NRF2 inhibitor KEAP1 (thus also known as INRF2) was unexpected; constitutive activation of NRF2 signaling leads to postnatal lethality [11]. Although *Keap1*-deficient mice exhibit signs of robust phase II detoxification, uncontrolled orthokeratinization obstructs the esophagus/forestomach and leads to malnutrition [11]. This unexpected phenotype remains a very important lesson for keratinocyte biologists [12]; however, the forced epidermal expression of the mutant *Nrf2* that lacks the KEAP1-interacting domain (ΔNeh2-*Nrf2*) [53] did not reproduce the stenosis of the upper digestive tract [17,53]. In contrast to humans, mice have a well-developed forestomach covered with keratinizing stratified squamous epithelium [54]. Despite being at the inner wet surface, the murine forestomach expresses LOR and could mimic the epidermis [55]. The evidence thus leads to the following conclusions: (i) The KEAP1/NRF2 signaling pathway does not affect keratinocyte differentiation but primarily alters adaptive responses following epidermal development [17,53]; (ii) ARCI-like hyperkeratosis likely reflects excessive antioxidative responses that produce thiol-rich proteins, which are then subjected to autooxidation upon exposure to ambient air in the SG [11,17,53,56,57]; (iii) Because NRF2 protects against hemodynamic shear stress [58], the keratinized obstruction of the digestive tract could be a consequence of peristaltic movement, which produces intermittent mechanical loads that could be sensed by cysteine residues on KEAP1 [11,12].

**Figure 2:**
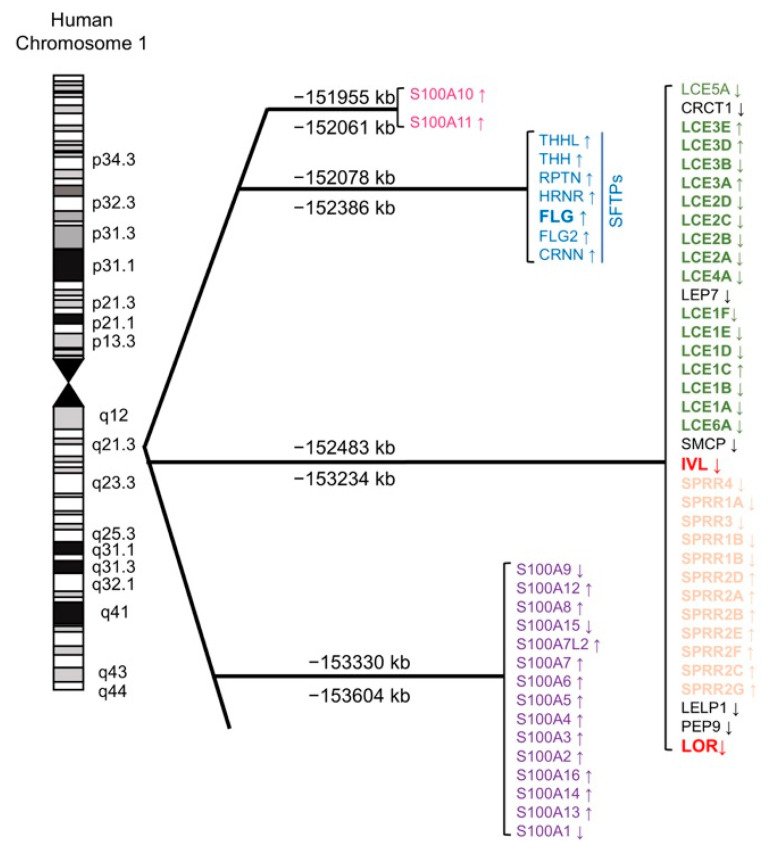
The epidermal differentiation complex (EDC). Schematic representation of human chromosome 1 and the EDC genes, which are thought to have evolved from a common ancestor and constitute diversified subfamilies.

**Figure 3:**
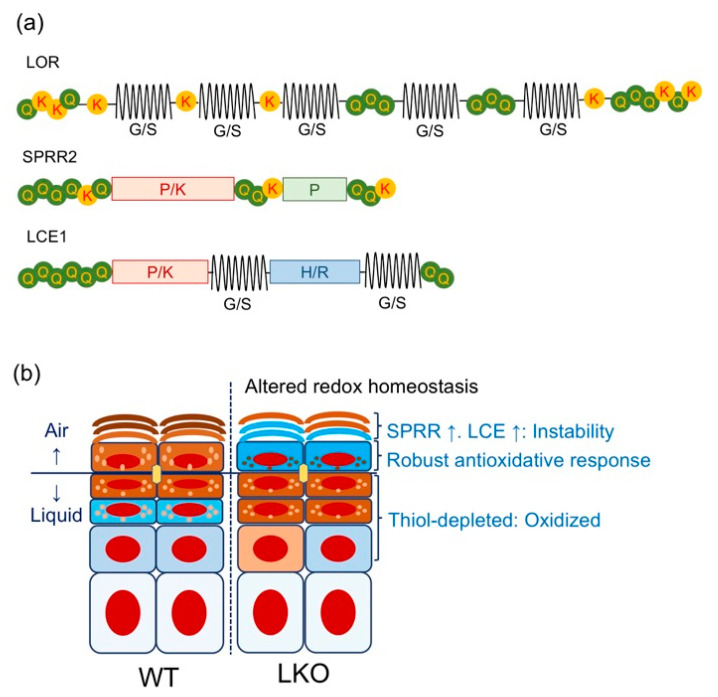
(**a**) The primary structure of murine LOR, SPRR2, and LCE1. LOR is a major CE component that contains high amounts of glycine (G; 55%), serine (S; 22%), and cysteine (C; 7%) arranged in tandem peptide quasi-repeats. These repeats are separated by highly conserved lysine (K) and glutamine (Q) residues that are substrates for transglutaminase-mediated ε-(γ-glutamyl) lysine cross-linkage. The consensus sequence of the SPRR2 family comprises almost entirely of proline (P), Q, K, and C. The consensus sequence of the LCE1 family suggests high amounts of P (~12%), as well as G (~20%) and S (~25%), suggesting that LCE1s have evolved as hybrids between LOR and SPRR2s. The H/R-rich region is reminiscent of FLG, which provides the precursor of natural moisturizing factors. (**b**) Schematic representation of altered redox homeostasis in LKO epidermis.

Having defined the KEAP1/NRF2 signaling pathway as a regulator of keratinization, we will now discuss the EDC gene cluster, which spans 1.6 Mb of human chromosome 1q21 (mouse 3q) (Figure 2) [59]. The EDC contains three gene clusters encoding (i) CE precursor proteins IVL, LOR, small proline-rich proteins (SPRRs), and the late cornified envelope (LCE); (ii) S100 calcium-binding proteins containing EF-hand domains; (iii) and the “fused gene” proteins [S100 fused type proteins (SFTP)], such as FLG, hornerin, and repetin that evolved from (i) and (ii) [60]. Analogous to the β-globin gene cluster [61], CE precursor genes are considered to have evolved from common ancestors [62]. The major CE precursors *Ivl* and *Lor* share structural similarities. The C- and N-terminal domains of the EDC gene products have characteristic arrangements of glutamine and lysine residues, which are required for the formation of powerful ε-(γ-glutamyl) lysine covalent cross-linkages in the CE [62]. In contrast to the conserved terminal domain features, internal domains constitute the modern fragments and thus reflect adaptive gene evolution [62,63,64]. For instance, the bulk sequence of *Lor* harbors many tandem peptide quasi-repeats of aliphatic (glycine/serine/cysteine)*_n_*, which differ markedly in size and sequence between the mouse and human homologs (Figure 3a) [37]. Similar genetic divergences are noted in *Ivl*, which shows substantial variance even among the hominoids, such as the human and the gorilla [61]. Finally, recent phylogenetic evidence suggests that: (i) *Lor* emerged from a common ancestor of modern amniotes during the acquisition of a fully terrestrial lifestyle [32] and (ii) cetaceans such as whales or dolphins lost *Flg* [65] or caspase 14 [66], an FLG-degrading enzyme that produces the natural moisturizing factors in the corneocyte [67]. These deletions were likely advantageous to adapt the skin to an aquatic lifestyle. This evidence corroborates the notion that the EDC gene cluster is one of the most rapidly evolving genetic loci among terrestrial vertebrates [68]. Therefore, analogous to the β-globin gene cluster [61], to learn the evolution of the EDC gene cluster is to acknowledge the history of repeated attempts of environmental adaptation in the animal kingdom [39].

### 3.2. Lessons from Lor-Deficient Mice: Quasi-Normalcy is not Synonymous with Normalcy

The development of the SC permeability barrier is a highly ordered and patterned process [2]. The mammalian epidermal permeability barrier develops in the dorsal-to-ventral direction and coincides with LOR expression [69,70], suggesting that LOR is a vital SC component. However, *Lor*-knockout (LKO) mice exhibit a very mild phenotype [71], which was rather unexpected at that time [72]. Although corneocyte maturation (cytoskeletal cross-linkage; the brick) is impaired [36,71,73], loss of the major CE protein does not cause desiccation (caused by a leaky “mortar”) or adaptive hyperkeratosis that is the hallmark feature of a defective barrier, i.e., a cracked “mortar” [4,6,74]. Instead, simply stated, the absence of a heavily thiolated epidermal component in the uppermost living layer presumably causes redox imbalance and activates the KEAP1/NRF2 signaling pathway (Figure 3b) [14,15].

Overexpressed *Lor* in *Keap1*-null mice led us to investigate the upstream 5’ regions of CE precursor genes [11], and functional AREs were found in the putative promoter regions [11,14,15]. The NRF2-mediated adaptive stress response upregulates *Sprr2*s [14,71] and *Lce1*s [14], which are structurally related to LOR in terms of amino acid composition or predicted protein structure (Figure 3a) [14]. These “alternative” CE precursors are small in terms of molecular weight (around 10 kDa) and they are robustly induced during stress conditions such as ultraviolet (UV) B irradiation [75,76], wound healing [77], or tape-stripping [78]. Thus, LKO SC, in which these kinds of LOR “mimicry” are abundant [14,15,55,71], appears to be under a kind of constitutive oxidative injury, and the KEAP1/NRF2 signaling pathway is major backup machinery [14,15] behind the very mild, “quasi-normal” [55] skin phenotype.

Experiments in LKO mice have led to the following three conclusions [20]:

(i) LOR is dispensable for the lipid-based epidermal permeability barrier [69,71], in contrast to the earlier CE components [79].

(ii) LOR serves as an adapter that organizes substrates for ε-(γ-glutamyl) lysine cross-linkages in the CE [35,36].

(iii) LOR expression appears to constitute a major epidermal antioxidative response that protects against a range of xenobiotic insults [20].

## 4. The KEAP1/NRF2 Signaling Pathway and Epidermal Homeostasis

Other than the phase II detoxification [48] or keratinization [11], the KEAP1/NRF2 signaling pathway contributes to a myriad of environmental responses [13]. Although no apparent developmental defects have been noted in association with germline *Nrf2*-null mutations in mice [48] or humans (OMIM *600492), *Nrf2*-null mice remain an attractive mouse model to understand the NRF2-mediated biological responses [48]. Conversely, it is becoming clear that aberrantly activated NRF2 signaling pathway can also be toxic to humans [80], and particular attention has been paid in the field of cancer research [81]. Moreover, *Cre*-mediated gene deletion in mice enabled us and others to circumvent the lethal phenotype resulting from the germline *Keap1*-null mutation [11,82] and dissect the consequence of the NRF2 activation in a cell lineage-specific manner [83]. Here we (i) summarize the structural and functional basis of the KEAP1/NRF2 signaling pathway [13]; (ii) revisit the principle of keratinization focusing on sulfhydryl (-SH) and disulfide (-S-S-) [19], in connection with the thiol-based sensor-effector apparatus [13]; (iii) review the findings from mouse models in which the KEAP1/NRF2 signaling pathway is genetically manipulated with a particular focus on the epidermis.

### 4.1. Structural and Functional Basis of the KEAP1/NRF2 System: Form Follows Function

**Figure 4:**
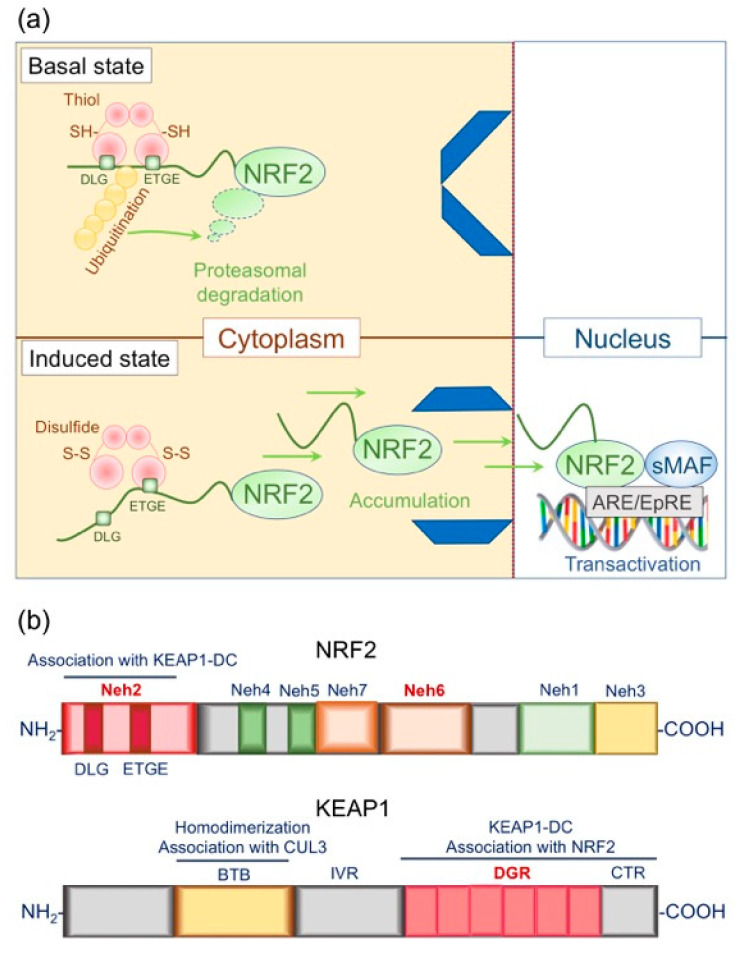
The structural and functional basis of the KEAP1/NRF2 signaling pathway. (**a**) The basics of the KEAP1/NRF2 signaling pathway. In the basal state, NRF2 is subjected to constant ubiquitin-proteasome-mediated proteolysis. NRF2 forms a cytosolic complex with KEAP1 via the DLG and ETGE motifs. In the induced state, modification of cysteine residues in KEAP1 causes a conformational change and liberates NRF2 from constant degradation, leading to nuclear accumulation and transactivation. (**b**) Schematic representation of the primary structure of NRF2 and KEAP1. Representative functions are indicated.

In the late 1990s, Ito et al. noticed that the canonical binding sequences of bZIP TFs were somewhat similar to the antioxidant responsive element (ARE) (also termed the electrophile response element, EpRE) [84,85], which is a critical *cis*-element of phase II detoxifying gene expression regulation [48]. This deduction led to the landmark discovery of KEAP1, which specifically binds with NRF2 and acts as a cytoplasmic inhibitor [50]. In steady-state conditions, NRF2 is constitutively poly-ubiquitinated by the cullin 3 (CUL3)-KEAP1 E3 ubiquitin ligase complex and subjected to proteasome-mediated degradation [13]. Electrophilic assaults or other redox-disruptive stimuli cause conformational changes in KEAP1, thus blocking the breakdown of NRF2 (Figure 4A) [13,49].

#### 4.1.1. Structural Basis of NRF2

The TF NRF2 is a member of the cap´n´collar (CNC) family. Initially, Ito et al. characterized six conserved domains with distinctive functions, Nrf2-erythroid cell-derived protein with CNC homology 1 (Neh1) to Neh6 in 1999 [49] (Figure 4B). Neh1 is the DNA-binding domain that interacts with the MAF bZIP TF, a heterodimerization partner of NRF2 [13]. The C-terminal Neh3 interacts with chromodomain helicase DNA-binding protein 6 and enhances the expression of a prototypical NRF2 target gene NAD(P)H quinone dehydrogenase 1 (*NQO1*) [86]. The Neh4/Neh5 complex interacts with CREB binding protein [87] or ectodermal-neural cortex 1 [88] and cooperatively transactivates NRF2 target genes. Conversely, the newly described Neh7, which abuts Neh5, directly interacts with retinoid X receptor alpha and negatively regulates ARE-mediated NRF2 target gene transcription [89]. The Neh2 and Neh6 domains are degrons that regulate proteasomal degradation of NRF2 protein [90]. Neh2 directly interacts with the KEAP1-double glycine repeat (DGR) and subjects NRF2 to proteasome-mediated degradation [13,49], while Neh6 mediates KEAP1-independent NRF2 degradation [90]. Glycogen synthase kinase 3 beta-mediated phosphorylation causes S-phase kinase-associated protein 1-cullin 1-ring-box 1 (SCF complex)-mediated proteasomal degradation of NRF2, thus attenuating excessive KEAP1/NRF2 signaling [91].

#### 4.1.2. Structural Basis of KEAP1

In the late 1990s, Ito et al. searched for a negative effector of NRF2 that specifically interacts with the Neh2 domain by using the yeast two-hybrid system [49]. Having screened 300 recovered clones, they identified a single protein with two evolutionarily conserved canonical protein interaction motifs, BTB (for the Broad-complex, Tramtrack, and Bric-à-brac) and the DGR. Since this motif combination is a characteristic feature of *Drosophila* cytoskeleton binding protein KELCH, they named the protein KEAP1 (Kelch-like erythroid cell-derived protein with CNC homology-associated protein 1) [49]. Because electrophilic stimuli disrupt the KEAP1-NRF2 interaction and activate phase II detoxification [49] an in-depth analysis of the signaling pathway could uncover the long-sought mechanism of the environmental response: the electrophilic counterattack response [92].

What serves as the molecular sensor of the electrophilic attack? In the early 2000s, Dinkova-Kostova et al. analyzed the structural and biochemical bases of KEAP1 in detail [93]. They looked for nucleophilic cysteine residues in the amino acid sequence of the NRF2 protein. However, the KEAP1-interacting Neh2 domain does not contain cysteine residues, and the NRF2 cysteine residues could not mediate the electrophile-induced signaling [93]. Murine KEAP1 contains 25 cysteines that are conserved in human and rat homologs [49] and encompasses five distinctive domains: the N-terminal region, the BTB, the intervening region (IVR), the DGR (also known as the Kelch repeat), and the C-terminal region [93], the latter two of which are called the KEAP1-DC complex [94] (Figure 4b). Although the initial attempt to purify KEAP1 protein resulted in the formation of disulfide cross-linked insoluble inclusion bodies, the most reactive cysteine residues were eventually mapped by electrophilic modification and mass spectrometric analysis of tryptic peptides [94]. After that, the substrate specificity of critical KEAP1 cysteine residues was identified as Cys151 in the BTB and Cys273/Cys288 in the IVR [13]. According to Dinkova-Kostova et al., there are three primary reactions: (i) The reaction of a single inducer molecule with two cysteine residues that are in spatial proximity. (ii) The reaction of a disulfide with a single cysteine, followed by an attack of another cysteine thiolate ion to form a disulfide bridge. Direct oxidation may have the same outcome. (iii) Irreversible reaction with an inducer leading to cysteine alkylation. These cysteine modifications cause conformational changes in KEAP1, resulting in partial dissociation of the KEAP1-NRF2 complex in the cytosol [93]. KEAP1/NRF2 signaling primarily mediates keratinization, as the thiol-rich KEAP1 protein senses the external oxidative milieu [49] and may inhibit keratinization [11].

KEAP1 forms a complex with the CUL3-rinx-box 1 holoenzyme through the BTB domain and thus serves as a substrate adaptor for the E3 ubiquitin ligase [13,95]. The BTB domain is required for KEAP1 homodimerization analogously to other proteins with the BTB motif, which led to the proposal of a two-site interaction model for the NRF2-KEAP1 complex [96,97,98]. Later, single-particle electron microscopy revealed two large spheres attached by short linker arms to the sides of a small forked-stem structure that resembles a cherry-bob, further supporting the model [99] (Figure 4a).

#### 4.1.3. Functional Basis of the KEAP1/NRF2 Signaling Pathway: Principles of Action

The constant mechanical load in the squamous epithelium and the disrupted NRF2-KEAP1 complex likely lead to excessive hyperkeratosis in *Keap1*-deficient mice [11,12,58]. Given that the mechanical stress perturbs cytoskeletal integrity, the KEAP1 protein, which is laden with labile thiol residues, may serve as a sensor for cytoskeletal dysregulation by internal or external causes [100]. At the same time, innate immunity may be inherently promiscuous [101]; the pattern recognition receptors not only recognize microorganismal components (such as nucleic acids, polysaccharides, or heat-shock proteins) but also respond to damaged, phylogenetically conserved “self” elements (damage-associated molecular patterns) [102,103]. Therefore, it would be tempting to speculate that, even more than the electrophilic counterattack response [92], the KEAP1/NRF2 signaling system evolved to facilitate various forms of prompt tissue responses. Thus, lethal hyperkeratosis in *Keap1*-deficient mice [11] or *Nrf2*-mediated epidermal barrier repair in LKO mice [14,15] are important examples of such adaptive responses in the epidermis. The functional understanding of KEAP1/NRF2 signaling in comparison with other gene expression regulation systems reveals the particularly “sensitive” nature of the thiol-based sensor-effector apparatus [13].

The above theoretical framework leads to important questions regarding the regulation of the onset and intensity of gene expression regulation. Changes in subcellular compartmentalization, i.e., from the cytosol to the nucleus, warrant subsequent transcriptional activation. One of the historic biochemical discoveries regarding the subcellular compartmentalization is the cytosolic complex of nuclear factor κB (NF-κB) and its inhibitor NF-κB inhibitor alpha (IκBα). In the late 1980s, cell fractionalization by Baltimore et al. revealed that cytosolic NF-κB possesses DNA-binding activity [104]. Analogous to the NRF2/KEAP1 complex, the dissociation of cytoplasmic NF-kB-IκBα complex liberates NF-kB DNA-binding activity [105]. Like other TFs, the effector protein NRF2 is primed in the cytosol, thus facilitating a prompt cellular response.

Under stressed conditions, NRF2 itself undergoes autoregulation through an ARE in its promoter and is thereby primed for further cellular distress [106]. However, as observed in the cell cycle, the phytochrome, and cell surface receptors, post-transcriptional regulation, such as phosphorylation or proteolysis, is common for TF-driven environmental responses in tissue. Protein kinase C-mediated phosphorylation of NRF2 is involved in ARE-mediated transactivation of phase II detoxifying enzyme *NQO1* [107,108]. However, as exemplified by other gene expression regulation systems, ubiquitin-proteasome proteolysis dominates the regulation of both TFs and their *trans*-acting factors [109], such as hypoxia-inducible factor 1 alpha (HIF-1α) [110] or tumor protein p53 (TP53) [111], and NFκB inhibitor IκBα [105]. The ubiquitin-proteasome proteolytic pathway constitutes the core element that facilitates successful TF-mediated tissue responses [109].

In steady-state conditions, NRF2 undergoes rapid turnover, while an oxidative milieu disrupts the interaction with KEAP1 and stabilizes NRF2 [112,113] (Figure 4a). This redox control of proteolysis and cytoplasmic-nuclear shuttling of the NRF2 protein depends on direct interaction with the KEAP1-DC [13]. The Neh2 domain contains two distinct conserved motifs: ETGE [114] and DLG [115] (Figure 4B). As in the two-site interaction models [96,97,116], ETGE and DLG each interact with one of the two KEAP1-DC domains in homodimerized KEAP1 protein [114,115]. Isothermal calorimetry revealed that compared with DLG, ETGE possesses a stronger binding affinity for KEAP1-DC [97,98]. In addition, DLG interacts rather loosely with KEAP1-DC and enables fast-on and fast-off binding, while the interaction of ETGE with KEAP1-DC better fits the two-state reaction model and ensures a tight binding that could facilitate the adaptor function of KEAP1 to the E3 ubiquitin ligase [117]. The DLG motif in the Neh2 degron may constitute an efficient sensor machinery that easily dissociates from KEAP1-DC [13,117]. Altogether, this evidence could explain why ETGE and DLG serve as “the hinge and the latch,” respectively [97,98].

#### 4.1.4. What Makes the KEAP1/NRF2 Signaling Pathway Sensitive and Efficient?

(i) A phylogenetically conserved interaction between KEAP1-DC and NRF2-Neh2 characterizes the essential nature of the KEAP1/NRF2 signaling pathway [49,114,115].

(ii) KEAP1 is the adaptor for the CUL3-based E3 ubiquitin ligase and subjects NRF2 to steady-state proteasomal degradation [95,112,113,118].

(iii) Conformational changes in the thiol-rich KEAP1 protein in response to redox-disruptive stimuli inhibit cytosolic NRF2 proteolysis and subsequently promote NRF2 shuttling to the nucleus [93,96,97,116].

(iv) For transcriptional activation, other proteasomal substrates such as IκBα [105] or HIF-1α [110], require IκB kinase-mediated phosphorylation [119] and O_2_-regulated proline hydroxylation [120], respectively. However, the proteasomal substrate NRF2 does not require such posttranslational modification but exclusively depends on the protein-protein interaction [98].

These characteristics likely allow the KEAP1/NRF2 signaling pathway to achieve incomparably sensitive and efficient environmental responses in multiple organ systems.

### 4.2. Nrf2 and Epidermal Barrier Function: Maintaining Epidermal Redox Balance

Normal keratinization requires the major thiol-rich CE protein LOR, which stabilizes corneocytes and protects against redox-disruptive assaults, such as UVB [73] or 7,12- dimethylbenz(a)anthracene (DMBA) [121]. Loss of LOR evokes NRF2-mediated adaptive stress responses, and LKO corneocytes are abundant in keratinocyte antioxidant SPRR2s [14,15] (Figure 3b). Because the extrusion of LG-derived lipid bilayers precedes LOR cross-linking into the CE in epidermal barrier development [69], it appears that the presence or absence of LOR does not interfere with the development of the lipid-based SC permeability barrier [69]. Therefore, it would be reasonable to formulate that LOR is indispensable for corneocyte maturation but dispensable for the lipid permeability barrier [71]. Thus, we now revisit the thiol (-SH)-disulfide (-S-S-) exchange in keratinization [19], which is the essential biochemical property that stabilizes the “brick” corneocyte [2].

It has been long known that the uptake of radiolabeled cystine (disulfide cross-linked cysteine) in the SG is near twice as much as that in the basal or the spinous layer [23,24]. Likewise, although urea-soluble living layer keratinocytes undergo a robust turnover of exogenous cystine, urea-insoluble terminally differentiated keratinocytes exhibit durable incorporation [25], thus demonstrating “enrichment in sulfur” in keratinization [19]. These classic observations may be analogous to the regulation of NRF2 activity: interaction with the KEAP1-DC through the Neh2 degron permits a rapid turnover, while the disulfide-mediated conformational change in the cytosol allows NRF2 transactivation and promotes keratinization [11,13].

#### 4.2.1. Keap1-Deficient Mice: the Epidermal “Keaper” and Striker

**Figure 5:**
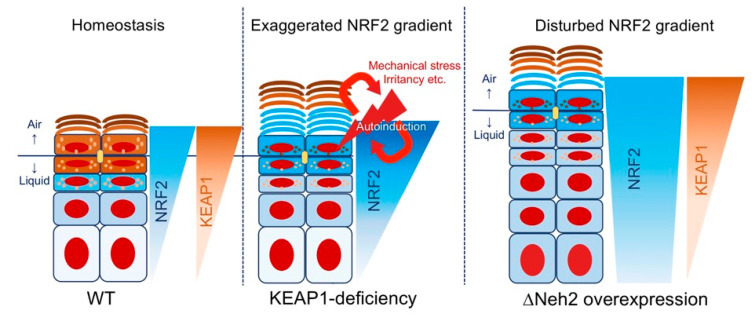
Why are *Keap1* deficiency and *Neh2* deficiency phenotypically different? Left: As with phase I detoxification, epidermal keratinocytes exhibit an incremented expression pattern of the KEAP1/NRF2 system that correlates with the degree of terminal differentiation. Middle: Absence of KEAP1 not only exaggerates the NRF2 expression gradient but also makes keratinocytes susceptible to cytoskeletal dysregulation (non-specific stimuli). Right: The overexpressed NRF2 that lacks the Neh2 degron augments NRF2 signaling in the presence of the inhibitor KEAP1. The disturbance in graded expression pattern in the epidermis mimics pathological conditions, such as ARCI or MADISH.

KEAP1 acts as a cytoplasmic molecular gatekeeper for NRF2 (Figure 4A); therefore, the absence of KEAP1 reduces the turnover of NRF2 protein and allows durable and potent cellular antioxidative responses [11]. Although *Nrf2*-deficient mice exhibit impaired induction of phase II detoxifying enzymes [48], it was surprising to witness the postnatal lethality of *Keap1*-deficient mice apparently caused by excessive keratinization of the esophagus/forestomach [11]. Hyperkeratinization of the inner squamous epithelium generated a large mass in the lumen that was palpable even from outside, and beneath the obstructing keratinizing mass was ulceration and inflammatory cell infiltrates [11]. Importantly, the proliferation of the squamous epithelium, as determined by the expression levels of proliferating cell nuclear antigen, was not affected, suggesting that *Keap1*-deficiency mediated NRF2 activation does not affect epidermal proliferation *per se* (Figure 5).

#### 4.2.2. Neh2-Nrf2 Mice: A Model of Gene-Environment Mismatch?

Schäfer and Werner et al. elaborated a series of mouse models in which *Nrf2* lacking the Nhe2 domain (ΔNeh2*-Nrf2*) is overexpressed in a tissue-specific manner [53]. This approach theoretically overcomes steady-state KEAP1-mediated NRF2 degradation and thus achieves constitutively activated NRF2 signaling [11,49]. After the development of the K5-cre recombinase-mediated overexpression model, they engineered the cytomegalovirus (CMV) enhancer/K5-cre-mediated overexpression model to examine if the ARCI-like phenotype [11,17] was NRF2 dose-dependent [17]. Indeed, the CMV/K5-cre-driven ΔNeh2*-Nrf2* (CMV/K5-ΔNeh2*-Nrf2*) overexpression reproduced the ARCI-like phenotype similar to *Keap1*-deficient mice [11,17], and topical application of *tert*-butylhydroquinone or sulforaphane phenocopied the skin manifestations [17].

Interestingly, aged CMV/K5-ΔNeh2*-Nrf2* mice exhibit multiple infundibular cysts highly reminiscent of chloracne/metabolizing acquired dioxin-induced skin hamartomas (MADISH) [18,53]. Because the polyaromatic hydrocarbon 2,3,7,8-tetrachlorodibenzo-p-dioxin (TCDD) activates the phase I xenometabolism, cystic hamartomas from MADISH patients express cytochrome P450 family 1 subfamily A member 1 (CYP1A1) [122] This critical clinical observation favors the notion that the MADISH is a consequence of persistent in situ activation of phase I detoxification [122], downstream of which is the KEAP1/NRF2 signaling [123]. Therefore, CMV/K5-ΔNeh2*-Nrf2* mice may indicate that locally activated xenobiotic metabolism underlies MADISH pathology [18]. Like other common ailments, gene-environment interaction [9] plays a significant role in acne vulgaris [124], which is caused by aberrant infundibular keratinization and sebum production [18,125]. Therefore, the observations from CMV/K5-ΔNeh2*-Nrf2* mice may support the notion that endogenous susceptibility factors [124], rather than exogenous disease modifiers, such as the cutaneous microbiome [126], can dominate the pathogenesis of common skin disorders.

#### 4.2.3. Summary: Why are Keap1 and Neh2 Deficiencies Phenotypically Different?

Having summarized mouse models in which the KEAP1/NRF2 signaling pathway is activated either by deleting the sensor [11] or by boosting the effector [53], we conclude that the differences between *Keap1* deficiency [11] and *Neh2* deficiency [53] would be attributable to (i) the exceptional efficiency of the thiol-rich sensor protein KEAP1, (ii) the ambient air-modification of keratinization, and (iii) the structural differences among thiol-rich CE precursors.

KEAP1 efficiency may be directly attributable to the phylogenetically conserved interaction between KEAP1-DC and NRF2-Neh2 [49,114,115]. The KEAP1/NRF2 signaling pathway does not require post-transcriptional modification but depends on labile cysteine residues, which would be suitable for detecting a myriad of non-specific stimuli, including biomechanical stresses [11,58] (Figure 5). Because the sensor is functional and serves as a gatekeeper in the ΔNeh2 models, the inner squamous epithelia are not affected, while epidermal hyperkeratosis is ubiquitous [11,16,17,18]. Epidermal hyperplasia [16,17,18] observed in the CMV/K5-ΔNeh2*-Nrf2* model could probably be attributable to NRF2 accumulation that overcame KEAP1-independent, phosphodegron Neh6/SCF-mediated proteasomal degradation [90,91], which theoretically affects cycling basal keratinocytes [127]. The perturbed NRF2 expression gradient may have led to delayed cell cycle exit and impaired disulfide cross-linkage formation [17] (Figure 5).

Although the inner squamous epithelia express LOR and thus mimic the process of epidermal differentiation, the keratinization of the inner epithelia appears very different from that in the epidermis. Keratinized materials in the wet surface squamous epithelium are exposed to oxidants, such as ozone or pollutants, to a much smaller degree than in the dry surface one. Exposure to the ambient air facilitates cross-linking while promoting keratin degradation induced by oxidants or UV [128,129] (Figure 1). Perhaps most importantly, as Steinert has put it, continued cross-linking in the SC can lead to the masking of the C-terminal peptide epitopes recognized by antibodies [34]. Indeed, LOR staining in the obstructed forestomach in *Keap1*-deficient mice looks retained even in the uppermost keratinized layer [11], while that in the SC decreases in the upper level, presumably in association with epitope masking [37]. In a similar vein, as Yoneda and Steinert et al. demonstrated earlier, human *Lor* overexpression in mice does not cause overt phenotypes, including in the esophagus/forestomach [130].

Given that the precipitation of thiolated proteins characterizes keratinization [19,23,24,25,26], the expression of NRF2 is probably hardwired into the epidermal differentiation program [53], and so is the thiol-rich protein KEAP1. This intrinsic gradient of KEAP1/NRF2 signaling not only facilitates keratinization but also may regulate uncontrolled hyperkeratinization, which altogether contributes to successful adaptation to the external environment [11]. Indeed, we recently found that high Ca^2+^ induces *KEAP1* expression in keratinocytes (Ogawa et al. manuscript submitted). In the differentiated layer, exposure to ambient air probably oxidizes cysteine residues and promotes the inhibition of Neh2-mediated NRF2 degradation, which further activates NRF2 signaling [11,17]. In the basal cycling layer, the Neh6 phosphodegron negatively regulates the intensity of NRF2 activation through SCF-mediated proteasomal degradation [90,91] primarily in the basal cycling keratinocytes [127].

Although ARE-mediated overexpression of *Lor* may be a cause of the luminal stenosis observed in *Keap1*-deficient mice [11], neither the ΔNeh2 mutation [17] nor tape-stripping [131,132] increased *Lor* expression levels in the epidermis. Thus, other contributing factors need to be considered [11,17]. Specifically, *Sprr2s* are keratinocyte antioxidants [77,133] downstream of NRF2 [15] that are also abundant in LKO cornified envelopes [15,71]. These kinds of LOR “mimicry” that cross-bridge various CE precursors [134] presumably compensate for the decreased cytoskeletal stability [135] or cytoskeletal redox imbalance [15] (Figure 3), both of which could be detected by the cytoplasmic sensor KEAP1. In this regard, Vermeij and Backendorf et al. elaborated on the superiority of SPRRs as antioxidants [77,133]. Nucleophilic cysteine residues characterize the antioxidative potential. However, as in the case of KEAP1 [93], due to the abundance of cysteines [27], the primary protein structure of LOR favors intra-/inter-molecular disulfide cross-linkages ex vivo [27,37] and hampers purification of crude LOR protein in an exogenous gene expression system [133]. In contrast, a sonicated CE, which is enriched in unlinked CE precursors such as SPRRs, rather than LOR [71], efficiently quenches reactive oxygen species [133]. Although this experimental setting may not be sufficient to assess the true antioxidative function of the SC in situ, the results indicate that the primary protein structure, irrespective of net cysteine content, determines the nucleophilic potential of thiol-rich CE precursors [133]. These crucial differences in the structure probably allow thiol-rich SPRR2 to remain nucleophilic in the ambient air, while LOR favors the inclusion/aggregation of the cellular content upon exposure to the ambient air [37], as was the case with KEAP1 [93]. In broad terms, the group of proteins with repetitive proline-rich sequences encompass salivary proline-rich proteins (PRPs) that efficiently bind polyphenols [136]. Therefore, the KEAP1/NRF2 signaling-mediated induction of SPRRs not only serves as a thiol-rich nucleophile [133] but may also take advantage of the adsorptive capacity of PRPs [137]. Binding of polyphenols to PRPs may further enhance antioxidative/detoxifying responses in the barrier tissue. However, as observed in LKO mice [71], the inherent reductive property of SPRR2s hampers disulfide cross-linkages and may have resulted in the accumulation of keratinized material on the inner surface [11], instead of thiol oxidation and disulfide cross-linkage-mediated stabilization [37]. Thus, the *Keap1*-deficient mouse phenotype may be regarded as an exaggerated form of reductive stress or maladaptation of the squamous epithelia [1,11]. Some have speculated that amniotes acquired LOR exclusively on the air-liquid interface to improve the fitness of the epidermal tissue because secreting antioxidative/detoxifying PRPs carries a high biological cost [15].

### 4.3. Epidermal Barrier Repair in LKO Mice

NRF2 mediates adaptive stress responses and upregulates “alternative” CE precursors, *Sprr2*s [15,71], or *Lce1*s [14], presumably in response to redox imbalance in the upper epidermis (Figure 3b). However, *Lor*/*Nrf2* double knockout mice do not show overt phenotypes, possibly due to the presence of other CNC family members, such as NRF1/NRF3 [138,139] or other *trans*-acting elements [140]. Therefore, we took a dominant-negative approach to overcome the compensation [138]. Specifically, we employed a transgenic mouse model that expresses dominant-negative *Nrf2* (dn*Nrf2*) under the control of the *Lor* promoter [15]. Introducing the transgene into LKO mice abrogated the compensatory response in utero and caused a delay in the formation of the permeability barrier as determined by a dye penetration assay [15,71]. An in vitro colorimetric reporter assay [15] showed that the increased electrophilic potential of amniotic fluid during the late gestational phases (embryonic day (E)14.5 vs. E16.5) serves as an environmental cue that activates the KEAP1/NRF2 signaling pathway [15]. These findings suggest that disulfide cross-linkage formation, which depends on the external oxidative milieu, is delayed in the absence of LOR. The “alternative” CE constituents, albeit potent nucleophiles [133], do not appear to stabilize the SC as efficiently as LOR [27,37]. Thiol oxidation in the SG promotes keratinization and thereby recovery of the epidermal permeability barrier when the living layer is exposed to the air-liquid interface [20]. In response to the external oxidative milieu, LOR mediates the formation of disulfide cross-linkages and may expedite the cooperative process of cytoskeletal cross-linkage (the brick) and the outside attachment of lipids (the mortar) to the outer surface of CEs [2,30].

### 4.4. NRF2 Activation and Epidermal Carcinogenesis: Friend or Foe?

Darwinian evolution embraces the core concept of gene-environment interaction, which can be applied to many biological behaviors [40], and carcinogenesis appears to be no exception [141]. In various cancers, somatic mutations in the NRF2-interacting KEAP1-DC [94] or the degron Neh2 [142] have been reported. These NRF2-activating mutations likely allow tumor cells to escape from the selective pressure in the microenvironment [141], presumably through increasing viability and reinforcing fitness.

Experimental evidence in the two-stage chemically-induced carcinogenesis setting could corroborate the above notion. The polyaromatic hydrocarbon DMBA undergoes CYP1A1/CYP1B1-mediated metabolic conversion that yields the ultimate mutagenic carcinogen DMBA-3,4-diol-1,2-epoxide (DMBADE) [143]. The “hard” electrophile DMBADE forms adducts with genomic DNA, while the cytosolic sensor protein KEAP1 activates the cytoprotective phase II detoxification pathway [13]. Thus, *Nrf2*-deficient mice were susceptible to chemically induced carcinogenesis, and the electrophilic counterattack response [92] requires the effector NRF2 [50]. Werner et al. demonstrated that the K14-dn*Nrf2* mice were less protected against chemically-induced carcinogenesis [138]. This study suggests that the cancer-protective effect of the KEAP1/NRF2 system is non-redundant [138]. Moreover, it is noteworthy that despite increased tumor burden, inhibition of NRF2-mediated gene expression does not largely affect the ensuing malignant conversion [papilloma to invasive squamous cell carcinoma (SCC)] [138]. Conversely, through the ΔNeh2*-Nrf2* transgenic approach [53], Schäfer et al. further elaborated on the keratinocyte NRF2 activation, which can be detrimental to the host [94], in two different cutaneous carcinogenesis settings. As expected, the keratinocyte-specific expression of the ΔNeh2*-Nrf2* protected against chemically induced carcinogenesis with reduced incidence rates of SCCs [16]. By contrast, when skin tumorigenesis was induced without exogenous carcinogen, ΔNeh2*-Nrf2* mice exhibited a tumor-promoting phenotype. Keratinocyte expression of early genes of human papillomavirus type 8 [144] along with the NRF2-activating mutation promoted skin tumorigenesis [16]. These lines of evidence suggest that constitutive activation of the KEAP1/NRF2 signaling pathway can permit tumor cells to withstand a harsh oxidative external microenvironment, delineating the “double-edged sword” aspect of the KEAP1/NRF2 signaling pathway [81]. This “dark side” of the cytoprotection machinery described as “NRF2 addiction” [145] may be analogous to what is caused by gain-of-function *TP53* mutations [146] in that the guardians of cellular fitness fuel malignant characteristics in tumor cells [147].

## 5. Concluding Remarks

**Figure 6:**
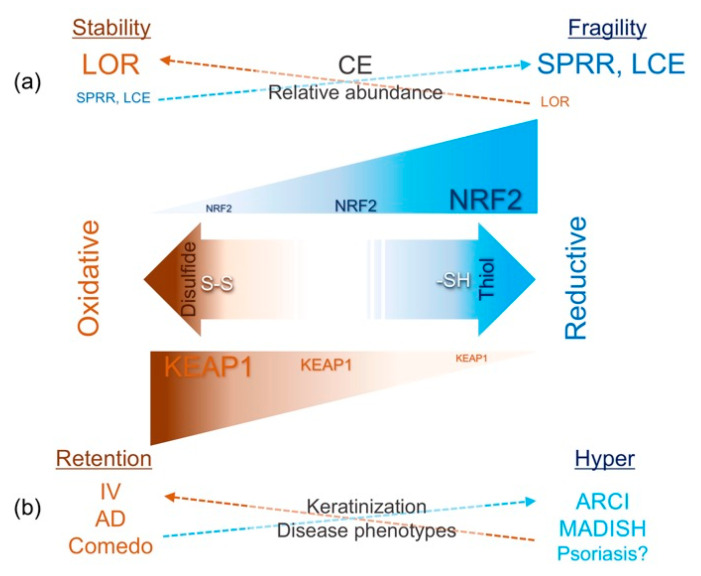
The KEAP1/NRF2 signaling pathway in keratinization and related skin diseases. (**a**) Phenotypes of the cornified cell envelope (CE). The oxidized status preferentially stabilizes the CE, and loricrin (LOR) promotes cytoskeletal cross-linkage. NRF2 repairs a defective barrier, probably in response to a perturbed redox balance, whereas persistent NRF2 activation hampers the formation of disulfide cross-linkage and makes the CE fragile. (**b**) Keratinization and disease phenotypes. An oxidative environment favors retention keratosis, which can lead to common dry skin conditions such as atopic dermatitis (AD) (clinically non-inflamed skin), or comedo (occluded hair follicle due to infundibular keratinization). On the other hand, strong and persistent NRF2 activation may be associated with autosomal recessive congenital ichthyosis (ARCI) or metabolizing acquired dioxin-induced skin hamartomas (MADISH).

We have reviewed the KEAP1/NRF2 signaling pathway with a special focus on the epidermis. As Marks and Plewig put it, the formation of the SC barrier is regarded as the successful adaptation of the epidermal tissue [1]. As observed in LKO mice [15] or psoriatic epidermis [56], proper activation of NRF2 signaling promotes the recovery of the epidermal permeability barrier, and regulates concurrent inflammatory responses (Figure 6). However, improper activation of the epidermal antioxidative responses leads to various kinds of skin disorders, such as acne [18] or AD [148] (Figure 6). Therefore, the thiol-based sensor-effector apparatus [13] constitutes an essential gatekeeper of epidermal homeostasis. Intriguingly, the autophagy cargo receptor sequestosome 1 (SQSTM1 or p62) directly interacts with KEAP1 and competitively inhibits its interaction with NRF2 at the DLG [149], which constitutes the “loose end” in the two-site KEAP1-NRF2 interaction model [96,97,98]. Indeed, in a manner analogous to the KEAP1-NRF2 interaction [49,114,115], the autophagy machinery is regarded as an evolutionally conserved stress response that allows unicellular eukaryotic organisms to survive during harsh conditions through recycling energy [150]. As morphological observations by Lavker and Matoltsy indicated half a century ago, keratinocyte transition from the SG to the SC involves lysosomal degradation of organelles [29], suggesting that autophagy is involved in keratinization [151]. Furthermore, p62 targets intracellular bacteria/viruses [152], but impaired autophagic clearance of pathogens, i.e., xenophagy [150], leads to cytoplasmic inclusion bodies [153]. This suggests that common viral infections of the skin, such as herpes simplex or molluscum contagiosum, may be associated with local deregulation of xenophagy [149,152]. Conversely, AD pathology involves broad defects in epidermal differentiation [153] and frequent skin infections by intracellular pathogens [154]. As indicated by the immunogenicity of the SC [155,156], homeostatic keratinization not only provides physical insulation but may also constitute an innate immune defense. Therefore, investigating the p62-KEAP1-mediated activation of NRF2 signaling [149] in the epidermis may uncover previously unappreciated aspects of keratinization and inflammatory/infectious skin conditions. Intensive investigation of this attractive research field may pave a way to mechanism-based and target-selective therapeutic measures in dermatology.

## Abbreviations

AD	atopic dermatitis
ARCI	autosomal recessive congenital ichthyosis
ARE	antioxidant response element
ARE	antioxidant responsive element
BTB	broad-complex, tramtrack, and bric-à-brac
bZIP	basic leucine zipper
CE	cornified cell envelope
CMV	cytomegalovirus
CNC	cap ´n´ collar
CTR	C-terminal region
CUL3	cullin 3
CYP1A1	cytochrome P450 family 1 subfamily A member 1
DGR	double glycine repeat
DMBA	7,12- dimethylbenz(a)anthracene
DMBADE	DMBA-3,4-diol-1,2-epoxide
ΔNeh2-Nrf2	Nrf2 that lacks KEAP1-interacting domain
dnNrf2	dominant-negative Nrf2
E	embryonic day
EDC	epidermal differentiation complex
EpRE	electrophile response element
FLG	filaggrin
GATA1	GATA protein binding protein 1
HIF-1α	hypoxia inducible factor 1 alpha
IκBα	NF-κB inhibitor alpha
IVL	involucrin
KEAP1	Kelch-like erythroid cell-derived protein with cap´n´collar homology-associated protein 1
KEAP1-DC	KEAP1 double glycine repeat and the C-terminal region
LCE	late cornified envelope
LG	lamellar granules
LKO	Lor-knockout (LKO)
LOR	loricrin
MADISH	metabolizing acquired dioxin-induced skin hamartomas
Neh	Nrf2-erythroid cell-derived protein with CNC homology
NF-κB	nuclear factor κB
NFE2	nuclear factor erythroid 2
NQO1	NAD(P)H quinone dehydrogenase 1
NRF2	NFE2-related factor 2
PRP	proline-rich proteins
S100	S100 calcium-binding proteins
SC	stratum corneum
SCC	squamous cell carcinoma
SCF	complex S-phase kinase associated protein 1-cullin 1-ring-box 1
SG	stratum granulosum
SPRR	small proline-rich protein
SQSTM1	sequestosome 1
TCDD	2,3,7,8-tetrachlorodibenzo-p-dioxin
TF	transcription factor
TP53	tumor protein p53
UV	ultraviolet

## References

[1] Marks R., Plewig G. (2012). Stratum Corneum.

[2] Nemes Z., Steinert P.M. (1999). Bricks and mortar of the epidermal barrier. Exp. Mol. Med..

[3] Takeichi T., Akiyama M. (2016). Inherited ichthyosis: Non-syndromic forms. J. Dermatol..

[4] Zuo Y., Zhuang D.Z., Han R., Isaac G., Tobin J.J., McKee M., Welti R., Brissette J.L., Fitzgerald M.L., Freeman M.W. (2008). ABCA12 maintains the epidermal lipid permeability barrier by facilitating formation of ceramide linoleic esters. J. Biol. Chem..

[5] Yanagi T., Akiyama M., Nishihara H., Ishikawa J., Sakai K., Miyamura Y., Naoe A., Kitahara T., Tanaka S., Shimizu H. (2010). Self-improvement of keratinocyte differentiation defects during skin maturation in ABCA12-deficient harlequin ichthyosis model mice. Am. J. Pathol..

[6] Kuramoto N., Takizawa T., Takizawa T., Matsuki M., Morioka H., Robinson J.M., Yamanishi K. (2002). Development of ichthyosiform skin compensates for defective permeability barrier function in mice lacking transglutaminase 1. J. Clin. Investig..

[7] Palmer C.N., Irvine A.D., Terron-Kwiatkowski A., Zhao Y., Liao H., Lee S.P., Goudie D.R., Sandilands A., Campbell L.E., Smith F.J. (2006). Common loss-of-function variants of the epidermal barrier protein filaggrin are a major predisposing factor for atopic dermatitis. Nat. Genet..

[8] Paller A.S., Spergel J.M., Mina-Osorio P., Irvine A.D. (2019). The atopic march and atopic multimorbidity: Many trajectories, many pathways. J. Allergy Clin. Immunol..

[9] Lieberman D. (2014). The Story of the Human Body: Evolution, Health, and Disease.

[10] Jones C.L., Reiners J.J. (1997). Differentiation status of cultured murine keratinocytes modulates induction of genes responsive to 2,3,7,8-tetrachlorodibenzo-p-dioxin. Arch. Biochem. Biophys..

[11] Wakabayashi N., Itoh K., Wakabayashi J., Motohashi H., Noda S., Takahashi S., Imakado S., Kotsuji T., Otsuka F., Roop D.R. (2003). Keap1-null mutation leads to postnatal lethality due to constitutive Nrf2 activation. Nat. Genet..

[12] Magin T.M. (2003). A keaper and a striker maintain epidermal homeostasis. Nat. Genet..

[13] Yamamoto M., Kensler T.W., Motohashi H. (2018). The KEAP1-NRF2 System: A Thiol-Based Sensor-Effector Apparatus for Maintaining Redox Homeostasis. Physiol. Rev..

[14] Ishitsuka Y., Huebner A.J., Rice R.H., Koch P.J., Speransky V.V., Steven A.C., Roop D.R. (2016). Lce1 Family Members Are Nrf2-Target Genes that Are Induced to Compensate for the Loss of Loricrin. J. Investig. Dermatol..

[15] Huebner A.J., Dai D., Morasso M., Schmidt E.E., Schafer M., Werner S., Roop D.R. (2012). Amniotic fluid activates the nrf2/keap1 pathway to repair an epidermal barrier defect in utero. Dev. Cell.

[16] Rolfs F., Huber M., Kuehne A., Kramer S., Haertel E., Muzumdar S., Wagner J., Tanner Y., Bohm F., Smola S. (2015). Nrf2 Activation Promotes Keratinocyte Survival during Early Skin Carcinogenesis via Metabolic Alterations. Cancer Res..

[17] Schafer M., Farwanah H., Willrodt A.H., Huebner A.J., Sandhoff K., Roop D., Hohl D., Bloch W., Werner S. (2012). Nrf2 links epidermal barrier function with antioxidant defense. EMBO Mol. Med..

[18] Schafer M., Willrodt A.H., Kurinna S., Link A.S., Farwanah H., Geusau A., Gruber F., Sorg O., Huebner A.J., Roop D.R. (2014). Activation of Nrf2 in keratinocytes causes chloracne (MADISH)-like skin disease in mice. EMBO Mol. Med..

[19] Van Scott E., Flesch P. (1954). Sulfhydryl and disulfide in keratinization. Science.

[20] Ishitsuka Y., Roop D.R. (2020). Loricrin: Past, Present, and Future. Int. J. Mol. Sci..

[21] Rice R.H., Green H. (1977). The cornified envelope of terminally differentiated human epidermal keratinocytes consists of cross-linked protein. Cell.

[22] Rice R.H., Green H. (1979). Presence in human epidermal cells of a soluble protein precursor of the cross-linked envelope: Activation of the cross-linking by calcium ions. Cell.

[23] Bern H.A., Harkness D.R., Blair S.M. (1955). Radioautographic studies of keratin formation. Proc. Natl. Acad. Sci. USA.

[24] Fukuyama K., Epstein W.L. (1969). Sulfur-containing proteins and epidermal keratinization. J. Cell Biol..

[25] Tezuka T., Hirai R. (1980). The synthesis of the cystine-rich proteins in rat epidermis: I. Analysis by [35S]cystine incorporation. Curr. Probl. Dermatol..

[26] Tezuka T., Takahashi M. (1987). The cystine-rich envelope protein from human epidermal stratum corneum cells. J. Investig. Dermatol..

[27] Mehrel T., Hohl D., Rothnagel J.A., Longley M.A., Bundman D., Cheng C., Lichti U., Bisher M.E., Steven A.C., Steinert P.M. (1990). Identification of a major keratinocyte cell envelope protein, loricrin. Cell.

[28] Matsui T., Amagai M. (2015). Dissecting the formation, structure and barrier function of the stratum corneum. Int. Immunol..

[29] Lavker R.M., Matoltsy A.G. (1970). Formation of horny cells: The fate of cell organelles and differentiation products in ruminal epithelium. J. Cell Biol..

[30] Nemes Z., Marekov L.N., Fesus L., Steinert P.M. (1999). A novel function for transglutaminase 1: Attachment of long-chain omega-hydroxyceramides to involucrin by ester bond formation. Proc. Natl. Acad. Sci. USA.

[31] Meyer A., Zardoya R. (2003). Recent advances in the (molecular) phylogeny of vertebrates. Ann. Rev. Ecol. Evol. Syst..

[32] Strasser B., Mlitz V., Hermann M., Rice R.H., Eigenheer R.A., Alibardi L., Tschachler E., Eckhart L. (2014). Evolutionary origin and diversification of epidermal barrier proteins in amniotes. Mol. Biol. Evol..

[33] Steven A.C., Bisher M.E., Roop D.R., Steinert P.M. (1990). Biosynthetic pathways of filaggrin and loricrin--two major proteins expressed by terminally differentiated epidermal keratinocytes. J. Struct. Biol..

[34] Steinert P.M., Cantieri J.S., Teller D.C., Lonsdale-Eccles J.D., Dale B.A. (1981). Characterization of a class of cationic proteins that specifically interact with intermediate filaments. Proc. Natl. Acad. Sci. USA.

[35] Steinert P.M., Marekov L.N. (1995). The proteins elafin, filaggrin, keratin intermediate filaments, loricrin, and small proline-rich proteins 1 and 2 are isodipeptide cross-linked components of the human epidermal cornified cell envelope. J. Biol. Chem..

[36] Rice R.H., Durbin-Johnson B.P., Ishitsuka Y., Salemi M., Phinney B.S., Rocke D.M., Roop D.R. (2016). Proteomic Analysis of Loricrin Knockout Mouse Epidermis. J. Proteome Res..

[37] Hohl D., Mehrel T., Lichti U., Turner M.L., Roop D.R., Steinert P.M. (1991). Characterization of human loricrin. Structure and function of a new class of epidermal cell envelope proteins. J. Biol. Chem..

[38] Fay J.C., Wu C.I. (2000). Hitchhiking under positive Darwinian selection. Genetics.

[39] Dawkins R. (2016). The Selfish Gene.

[40] Choi O.R., Engel J.D. (1988). Developmental regulation of beta-globin gene switching. Cell.

[41] Natarajan C., Hoffmann F.G., Weber R.E., Fago A., Witt C.C., Storz J.F. (2016). Predictable convergence in hemoglobin function has unpredictable molecular underpinnings. Science.

[42] Taylor S.M., Parobek C.M., Fairhurst R.M. (2012). Haemoglobinopathies and the clinical epidemiology of malaria: A systematic review and meta-analysis. Lancet Infect. Dis..

[43] Moi P., Chan K., Asunis I., Cao A., Kan Y.W. (1994). Isolation of NF-E2-related factor 2 (Nrf2), a NF-E2-like basic leucine zipper transcriptional activator that binds to the tandem NF-E2/AP1 repeat of the beta-globin locus control region. Proc. Natl. Acad. Sci. USA.

[44] Itoh K., Igarashi K., Hayashi N., Nishizawa M., Yamamoto M. (1995). Cloning and characterization of a novel erythroid cell-derived CNC family transcription factor heterodimerizing with the small Maf family proteins. Mol. Cell. Biol..

[45] Pevny L., Simon M.C., Robertson E., Klein W.H., Tsai S.F., D’Agati V., Orkin S.H., Costantini F. (1991). Erythroid differentiation in chimaeric mice blocked by a targeted mutation in the gene for transcription factor GATA-1. Nature.

[46] Shivdasani R.A., Orkin S.H. (1995). Erythropoiesis and globin gene expression in mice lacking the transcription factor NF-E2. Proc. Natl. Acad. Sci. USA.

[47] Chan K., Lu R., Chang J.C., Kan Y.W. (1996). NRF2, a member of the NFE2 family of transcription factors, is not essential for murine erythropoiesis, growth, and development. Proc. Natl. Acad. Sci. USA.

[48] Itoh K., Chiba T., Takahashi S., Ishii T., Igarashi K., Katoh Y., Oyake T., Hayashi N., Satoh K., Hatayama I. (1997). An Nrf2/small Maf heterodimer mediates the induction of phase II detoxifying enzyme genes through antioxidant response elements. Biochem. Biophys. Res. Commun..

[49] Itoh K., Wakabayashi N., Katoh Y., Ishii T., Igarashi K., Engel J.D., Yamamoto M. (1999). Keap1 represses nuclear activation of antioxidant responsive elements by Nrf2 through binding to the amino-terminal Neh2 domain. Genes Dev..

[50] Kitamura Y., Umemura T., Kanki K., Kodama Y., Kitamoto S., Saito K., Itoh K., Yamamoto M., Masegi T., Nishikawa A. (2007). Increased susceptibility to hepatocarcinogenicity of Nrf2-deficient mice exposed to 2-amino-3-methylimidazo[4,5-f]quinoline. Cancer Sci..

[51] Ramos-Gomez M., Kwak M.K., Dolan P.M., Itoh K., Yamamoto M., Talalay P., Kensler T.W. (2001). Sensitivity to carcinogenesis is increased and chemoprotective efficacy of enzyme inducers is lost in nrf2 transcription factor-deficient mice. Proc. Natl. Acad. Sci. USA.

[52] Xu C., Huang M.T., Shen G., Yuan X., Lin W., Khor T.O., Conney A.H., Kong A.N. (2006). Inhibition of 7,12-dimethylbenz(a)anthracene-induced skin tumorigenesis in C57BL/6 mice by sulforaphane is mediated by nuclear factor E2-related factor 2. Cancer Res..

[53] Schafer M., Dutsch S., auf dem Keller U., Navid F., Schwarz A., Johnson D.A., Johnson J.A., Werner S. (2010). Nrf2 establishes a glutathione-mediated gradient of UVB cytoprotection in the epidermis. Genes Dev..

[54] Kim T.H., Shivdasani R.A. (2016). Stomach development, stem cells and disease. Development.

[55] Jarnik M., de Viragh P.A., Scharer E., Bundman D., Simon M.N., Roop D.R., Steven A.C. (2002). Quasi-normal cornified cell envelopes in loricrin knockout mice imply the existence of a loricrin backup system. J. Investig. Dermatol..

[56] Ogawa T., Ishitsuka Y., Inoue S., Nakamura Y., Saito A., Okiyama N., Fujisawa Y., Furuta J., Watanabe R., Fujimoto M. (2020). Nuclear Factor Erythroid 2-Related Factor 2 (Nrf2) Regulates Epidermal Keratinization under Psoriatic Skin Inflammation. Am. J. Pathol..

[57] Redondo P., Bauza A. (1999). Topical N-acetylcysteine for lamellar ichthyosis. Lancet.

[58] Li J., Ichikawa T., Villacorta L., Janicki J.S., Brower G.L., Yamamoto M., Cui T. (2009). Nrf2 protects against maladaptive cardiac responses to hemodynamic stress. Arterioscler. Thromb. Vasc. Biol..

[59] Mischke D., Korge B.P., Marenholz I., Volz A., Ziegler A. (1996). Genes encoding structural proteins of epidermal cornification and S100 calcium-binding proteins form a gene complex (“epidermal differentiation complex”) on human chromosome 1q21. J. Investig. Dermatol.

[60] Kypriotou M., Huber M., Hohl D. (2012). The human epidermal differentiation complex: Cornified envelope precursors, S100 proteins and the ‘fused genes’ family. Exp. Dermatol..

[61] Efstratiadis A., Posakony J.W., Maniatis T., Lawn R.M., O’Connell C., Spritz R.A., DeRiel J.K., Forget B.G., Weissman S.M., Slightom J.L. (1980). The structure and evolution of the human beta-globin gene family. Cell.

[62] Backendorf C., Hohl D. (1992). A common origin for cornified envelope proteins?. Nat. Genet..

[63] Eckert R.L., Green H. (1986). Structure and evolution of the human involucrin gene. Cell.

[64] Teumer J., Green H. (1989). Divergent evolution of part of the involucrin gene in the hominoids: Unique intragenic duplications in the gorilla and human. Proc. Natl. Acad. Sci. USA.

[65] Oh J.W., Chung O., Cho Y.S., MacGregor G.R., Plikus M.V. (2015). Gene loss in keratinization programs accompanies adaptation of cetacean skin to aquatic lifestyle. Exp. Dermatol..

[66] Strasser B., Mlitz V., Fischer H., Tschachler E., Eckhart L. (2015). Comparative genomics reveals conservation of filaggrin and loss of caspase-14 in dolphins. Exp. Dermatol.

[67] Harding C.R., Aho S., Bosko C.A. (2013). Filaggrin-revisited. Int. J. Cosmet. Sci..

[68] Goodwin Z.A., de Guzman Strong C. (2016). Recent Positive Selection in Genes of the Mammalian Epidermal Differentiation Complex Locus. Front. Genet..

[69] Bickenbach J.R., Greer J.M., Bundman D.S., Rothnagel J.A., Roop D.R. (1995). Loricrin expression is coordinated with other epidermal proteins and the appearance of lipid lamellar granules in development. J. Investig. Dermatol..

[70] Hardman M.J., Sisi P., Banbury D.N., Byrne C. (1998). Patterned acquisition of skin barrier function during development. Development.

[71] Koch P.J., de Viragh P.A., Scharer E., Bundman D., Longley M.A., Bickenbach J., Kawachi Y., Suga Y., Zhou Z., Huber M. (2000). Lessons from loricrin-deficient mice: Compensatory mechanisms maintaining skin barrier function in the absence of a major cornified envelope protein. J. Cell Biol..

[72] Steinert P.M. (2000). The complexity and redundancy of epithelial barrier function. J. Cell Biol..

[73] Ishitsuka Y., Roop D.R. (2018). Loricrin Confers Photoprotective Function against UVB in Corneocytes. J. Investig. Dermatol..

[74] Yamanaka Y., Akiyama M., Sugiyama-Nakagiri Y., Sakai K., Goto M., McMillan J.R., Ota M., Sawamura D., Shimizu H. (2007). Expression of the keratinocyte lipid transporter ABCA12 in developing and reconstituted human epidermis. Am. J. Pathol..

[75] Gibbs S., Fijneman R., Wiegant J., van Kessel A.G., van De Putte P., Backendorf C. (1993). Molecular characterization and evolution of the SPRR family of keratinocyte differentiation markers encoding small proline-rich proteins. Genomics.

[76] Jackson B., Tilli C.M., Hardman M.J., Avilion A.A., MacLeod M.C., Ashcroft G.S., Byrne C. (2005). Late cornified envelope family in differentiating epithelia--response to calcium and ultraviolet irradiation. J. Investig. Dermatol..

[77] Vermeij W.P., Backendorf C. (2010). Skin cornification proteins provide global link between ROS detoxification and cell migration during wound healing. PLoS ONE.

[78] de Cid R., Riveira-Munoz E., Zeeuwen P.L., Robarge J., Liao W., Dannhauser E.N., Giardina E., Stuart P.E., Nair R., Helms C. (2009). Deletion of the late cornified envelope LCE3B and LCE3C genes as a susceptibility factor for psoriasis. Nat. Genet..

[79] Sevilla L.M., Nachat R., Groot K.R., Klement J.F., Uitto J., Djian P., Maatta A., Watt F.M. (2007). Mice deficient in involucrin, envoplakin, and periplakin have a defective epidermal barrier. J. Cell Biol..

[80] Huppke P., Weissbach S., Church J.A., Schnur R., Krusen M., Dreha-Kulaczewski S., Kuhn-Velten W.N., Wolf A., Huppke B., Millan F. (2017). Activating de novo mutations in NFE2L2 encoding NRF2 cause a multisystem disorder. Nat. Commun..

[81] Menegon S., Columbano A., Giordano S. (2016). The Dual Roles of NRF2 in Cancer. Trends Mol. Med..

[82] Taguchi K., Maher J.M., Suzuki T., Kawatani Y., Motohashi H., Yamamoto M. (2010). Genetic analysis of cytoprotective functions supported by graded expression of Keap1. Mol. Cell. Biol..

[83] Suzuki T., Murakami S., Biswal S.S., Sakaguchi S., Harigae H., Yamamoto M., Motohashi H. (2017). Systemic activation of NRF2 alleviates lethal autoimmune inflammation in scurfy mice. Mol. Cell. Biol..

[84] Rushmore T.H., Morton M.R., Pickett C.B. (1991). The antioxidant responsive element. Activation by oxidative stress and identification of the DNA consensus sequence required for functional activity. J. Biol. Chem..

[85] Wasserman W.W., Fahl W.E. (1997). Functional antioxidant responsive elements. Proc. Natl. Acad. Sci. USA.

[86] Nioi P., Nguyen T., Sherratt P.J., Pickett C.B. (2005). The carboxy-terminal Neh3 domain of Nrf2 is required for transcriptional activation. Mol. Cell. Biol..

[87] Katoh Y., Itoh K., Yoshida E., Miyagishi M., Fukamizu A., Yamamoto M. (2001). Two domains of Nrf2 cooperatively bind CBP, a CREB binding protein, and synergistically activate transcription. Genes Cells.

[88] Seng S., Avraham H.K., Jiang S., Yang S., Sekine M., Kimelman N., Li H., Avraham S. (2007). The nuclear matrix protein, NRP/B, enhances Nrf2-mediated oxidative stress responses in breast cancer cells. Cancer Res..

[89] Wang H., Liu K., Geng M., Gao P., Wu X., Hai Y., Li Y., Li Y., Luo L., Hayes J.D. (2013). RXRalpha inhibits the NRF2-ARE signaling pathway through a direct interaction with the Neh7 domain of NRF2. Cancer Res..

[90] McMahon M., Thomas N., Itoh K., Yamamoto M., Hayes J.D. (2004). Redox-regulated turnover of Nrf2 is determined by at least two separate protein domains, the redox-sensitive Neh2 degron and the redox-insensitive Neh6 degron. J. Biol. Chem..

[91] Chowdhry S., Zhang Y., McMahon M., Sutherland C., Cuadrado A., Hayes J.D. (2013). Nrf2 is controlled by two distinct beta-TrCP recognition motifs in its Neh6 domain, one of which can be modulated by GSK-3 activity. Oncogene.

[92] Prestera T., Zhang Y., Spencer S.R., Wilczak C.A., Talalay P. (1993). The electrophile counterattack response: Protection against neoplasia and toxicity. Adv. Enzym. Regul..

[93] Dinkova-Kostova A.T., Holtzclaw W.D., Cole R.N., Itoh K., Wakabayashi N., Katoh Y., Yamamoto M., Talalay P. (2002). Direct evidence that sulfhydryl groups of Keap1 are the sensors regulating induction of phase 2 enzymes that protect against carcinogens and oxidants. Proc. Natl. Acad. Sci. USA.

[94] Padmanabhan B., Tong K.I., Ohta T., Nakamura Y., Scharlock M., Ohtsuji M., Kang M.I., Kobayashi A., Yokoyama S., Yamamoto M. (2006). Structural basis for defects of Keap1 activity provoked by its point mutations in lung cancer. Mol. Cell.

[95] Cullinan S.B., Gordan J.D., Jin J., Harper J.W., Diehl J.A. (2004). The Keap1-BTB protein is an adaptor that bridges Nrf2 to a Cul3-based E3 ligase: Oxidative stress sensing by a Cul3-Keap1 ligase. Mol. Cell. Biol..

[96] McMahon M., Thomas N., Itoh K., Yamamoto M., Hayes J.D. (2006). Dimerization of substrate adaptors can facilitate cullin-mediated ubiquitylation of proteins by a “tethering” mechanism: A two-site interaction model for the Nrf2-Keap1 complex. J. Biol. Chem..

[97] Tong K.I., Katoh Y., Kusunoki H., Itoh K., Tanaka T., Yamamoto M. (2006). Keap1 recruits Neh2 through binding to ETGE and DLG motifs: Characterization of the two-site molecular recognition model. Mol. Cell. Biol..

[98] Tong K.I., Padmanabhan B., Kobayashi A., Shang C., Hirotsu Y., Yokoyama S., Yamamoto M. (2007). Different electrostatic potentials define ETGE and DLG motifs as hinge and latch in oxidative stress response. Mol. Cell. Biol..

[99] Ogura T., Tong K.I., Mio K., Maruyama Y., Kurokawa H., Sato C., Yamamoto M. (2010). Keap1 is a forked-stem dimer structure with two large spheres enclosing the intervening, double glycine repeat, and C-terminal domains. Proc. Natl. Acad. Sci. USA.

[100] Kang M.I., Kobayashi A., Wakabayashi N., Kim S.G., Yamamoto M. (2004). Scaffolding of Keap1 to the actin cytoskeleton controls the function of Nrf2 as key regulator of cytoprotective phase 2 genes. Proc. Natl. Acad. Sci. USA.

[101] Akira S., Takeda K. (2004). Toll-like receptor signalling. Nat. Rev. Immunol..

[102] Matzinger P. (2002). The danger model: A renewed sense of self. Science.

[103] Takeuchi O., Akira S. (2010). Pattern recognition receptors and inflammation. Cell.

[104] Baeuerle P.A., Baltimore D. (1988). Activation of DNA-binding activity in an apparently cytoplasmic precursor of the NF-kappa B transcription factor. Cell.

[105] Baeuerle P.A., Baltimore D. (1988). I kappa B: A specific inhibitor of the NF-kappa B transcription factor. Science.

[106] Kwak M.K., Itoh K., Yamamoto M., Kensler T.W. (2002). Enhanced expression of the transcription factor Nrf2 by cancer chemopreventive agents: Role of antioxidant response element-like sequences in the nrf2 promoter. Mol. Cell. Biol..

[107] Bloom D.A., Jaiswal A.K. (2003). Phosphorylation of Nrf2 at Ser40 by protein kinase C in response to antioxidants leads to the release of Nrf2 from INrf2, but is not required for Nrf2 stabilization/accumulation in the nucleus and transcriptional activation of antioxidant response element-mediated NAD(P)H:quinone oxidoreductase-1 gene expression. J. Biol. Chem..

[108] Huang H.C., Nguyen T., Pickett C.B. (2000). Regulation of the antioxidant response element by protein kinase C-mediated phosphorylation of NF-E2-related factor 2. Proc. Natl. Acad. Sci. USA.

[109] Ciechanover A. (1994). The ubiquitin-proteasome proteolytic pathway. Cell.

[110] Semenza G.L. (2007). Hypoxia-inducible factor 1 (HIF-1) pathway. Sci. STKE.

[111] Michael D., Oren M. (2003). The p53-Mdm2 module and the ubiquitin system. Semin. Cancer Biol..

[112] McMahon M., Itoh K., Yamamoto M., Hayes J.D. (2003). Keap1-dependent proteasomal degradation of transcription factor Nrf2 contributes to the negative regulation of antioxidant response element-driven gene expression. J. Biol. Chem..

[113] Nguyen T., Sherratt P.J., Huang H.C., Yang C.S., Pickett C.B. (2003). Increased protein stability as a mechanism that enhances Nrf2-mediated transcriptional activation of the antioxidant response element. Degradation of Nrf2 by the 26 S proteasome. J. Biol. Chem..

[114] Kobayashi M., Itoh K., Suzuki T., Osanai H., Nishikawa K., Katoh Y., Takagi Y., Yamamoto M. (2002). Identification of the interactive interface and phylogenic conservation of the Nrf2-Keap1 system. Genes Cells.

[115] Katoh Y., Iida K., Kang M.I., Kobayashi A., Mizukami M., Tong K.I., McMahon M., Hayes J.D., Itoh K., Yamamoto M. (2005). Evolutionary conserved N-terminal domain of Nrf2 is essential for the Keap1-mediated degradation of the protein by proteasome. Arch. Biochem. Biophys..

[116] Tong K.I., Kobayashi A., Katsuoka F., Yamamoto M. (2006). Two-site substrate recognition model for the Keap1-Nrf2 system: A hinge and latch mechanism. Biol. Chem..

[117] Fukutomi T., Takagi K., Mizushima T., Ohuchi N., Yamamoto M. (2014). Kinetic, thermodynamic, and structural characterizations of the association between Nrf2-DLGex degron and Keap1. Mol. Cell. Biol..

[118] Stewart D., Killeen E., Naquin R., Alam S., Alam J. (2003). Degradation of transcription factor Nrf2 via the ubiquitin-proteasome pathway and stabilization by cadmium. J. Biol. Chem..

[119] Zandi E., Rothwarf D.M., Delhase M., Hayakawa M., Karin M. (1997). The IkappaB kinase complex (IKK) contains two kinase subunits, IKKalpha and IKKbeta, necessary for IkappaB phosphorylation and NF-kappaB activation. Cell.

[120] Jaakkola P., Mole D.R., Tian Y.M., Wilson M.I., Gielbert J., Gaskell S.J., von Kriegsheim A., Hebestreit H.F., Mukherji M., Schofield C.J. (2001). Targeting of HIF-alpha to the von Hippel-Lindau ubiquitylation complex by O2-regulated prolyl hydroxylation. Science.

[121] Ogawa T., Ishitsuka Y., Roop D., Fujimoto M. (2019). 314 Loricrin protects against chemical carcinogenesis but affects cancer immunoediting. J. Investig. Dermatol..

[122] Saurat J.H., Kaya G., Saxer-Sekulic N., Pardo B., Becker M., Fontao L., Mottu F., Carraux P., Pham X.C., Barde C. (2012). The cutaneous lesions of dioxin exposure: Lessons from the poisoning of Victor Yushchenko. Toxicol. Sci..

[123] Klaassen C.D., Reisman S.A. (2010). Nrf2 the rescue: Effects of the antioxidative/electrophilic response on the liver. Toxicol. Appl. Pharmacol..

[124] Common J.E.A., Barker J.N., van Steensel M.A.M. (2019). What does acne genetics teach us about disease pathogenesis?. Br. J. Dermatol..

[125] Williams H.C., Dellavalle R.P., Garner S. (2012). Acne vulgaris. Lancet.

[126] Woo T.E., Sibley C.D. (2020). The emerging utility of the cutaneous microbiome in the treatment of acne and atopic dermatitis. J. Am. Acad. Dermatol..

[127] Vodermaier H.C. (2004). APC/C and SCF: Controlling each other and the cell cycle. Curr. Biol..

[128] Steinert P.M., Idler W.W. (1979). Postsynthetic modifications of mammalian epidermal alpha-keratin. Biochemistry.

[129] Thiele J.J., Hsieh S.N., Briviba K., Sies H. (1999). Protein oxidation in human stratum corneum: Susceptibility of keratins to oxidation in vitro and presence of a keratin oxidation gradient in vivo. J. Investig. Dermatol..

[130] Yoneda K., Steinert P.M. (1993). Overexpression of human loricrin in transgenic mice produces a normal phenotype. Proc. Natl. Acad. Sci. USA.

[131] de Koning H.D., van den Bogaard E.H., Bergboer J.G., Kamsteeg M., van Vlijmen-Willems I.M., Hitomi K., Henry J., Simon M., Takashita N., Ishida-Yamamoto A. (2012). Expression profile of cornified envelope structural proteins and keratinocyte differentiation-regulating proteins during skin barrier repair. Br. J. Dermatol..

[132] Ekanayake-Mudiyanselage S., Aschauer H., Schmook F.P., Jensen J.M., Meingassner J.G., Proksch E. (1998). Expression of epidermal keratins and the cornified envelope protein involucrin is influenced by permeability barrier disruption. J. Investig. Dermatol..

[133] Vermeij W.P., Alia A., Backendorf C. (2011). ROS quenching potential of the epidermal cornified cell envelope. J. Investig. Dermatol..

[134] Steinert P.M., Candi E., Kartasova T., Marekov L. (1998). Small proline-rich proteins are cross-bridging proteins in the cornified cell envelopes of stratified squamous epithelia. J. Struct. Biol..

[135] Steinert P.M., Kartasova T., Marekov L.N. (1998). Biochemical evidence that small proline-rich proteins and trichohyalin function in epithelia by modulation of the biomechanical properties of their cornified cell envelopes. J. Biol. Chem..

[136] Hagerman A.E., Butler L.G. (1981). The specificity of proanthocyanidin-protein interactions. J. Biol. Chem..

[137] Williamson M.P. (1994). The structure and function of proline-rich regions in proteins. Biochem. J..

[138] auf dem Keller U., Huber M., Beyer T.A., Kumin A., Siemes C., Braun S., Bugnon P., Mitropoulos V., Johnson D.A., Johnson J.A. (2006). Nrf transcription factors in keratinocytes are essential for skin tumor prevention but not for wound healing. Mol. Cell. Biol..

[139] Braun S., Hanselmann C., Gassmann M.G., auf dem Keller U., Born-Berclaz C., Chan K., Kan Y.W., Werner S. (2002). Nrf2 transcription factor, a novel target of keratinocyte growth factor action which regulates gene expression and inflammation in the healing skin wound. Mol. Cell. Biol..

[140] Fan X., Wang D., Burgmaier J.E., Teng Y., Romano R.A., Sinha S., Yi R. (2018). Single Cell and Open Chromatin Analysis Reveals Molecular Origin of Epidermal Cells of the Skin. Dev. Cell.

[141] Nowell P.C. (1976). The clonal evolution of tumor cell populations. Science.

[142] Shibata T., Ohta T., Tong K.I., Kokubu A., Odogawa R., Tsuta K., Asamura H., Yamamoto M., Hirohashi S. (2008). Cancer related mutations in NRF2 impair its recognition by Keap1-Cul3 E3 ligase and promote malignancy. Proc. Natl. Acad. Sci. USA.

[143] Miyata M., Kudo G., Lee Y.H., Yang T.J., Gelboin H.V., Fernandez-Salguero P., Kimura S., Gonzalez F.J. (1999). Targeted disruption of the microsomal epoxide hydrolase gene. Microsomal epoxide hydrolase is required for the carcinogenic activity of 7,12-dimethylbenz[a]anthracene. J. Biol. Chem..

[144] Schaper I.D., Marcuzzi G.P., Weissenborn S.J., Kasper H.U., Dries V., Smyth N., Fuchs P., Pfister H. (2005). Development of skin tumors in mice transgenic for early genes of human papillomavirus type 8. Cancer Res..

[145] Kitamura H., Motohashi H. (2018). NRF2 addiction in cancer cells. Cancer Sci..

[146] Terzian T., Suh Y.A., Iwakuma T., Post S.M., Neumann M., Lang G.A., Van Pelt C.S., Lozano G. (2008). The inherent instability of mutant p53 is alleviated by Mdm2 or p16INK4a loss. Genes Dev..

[147] Mantovani F., Collavin L., Del Sal G. (2019). Mutant p53 as a guardian of the cancer cell. Cell Death Differ..

[148] Ogawa T., Ishitsuka Y., Nakamura Y., Kubota N., Saito A., Fujisawa Y., Watanabe R., Okiyama N., Suga Y., Roop D.R. (2020). NRF2 Augments Epidermal Antioxidant Defenses and Promotes Atopy. J. Immunol..

[149] Komatsu M., Kurokawa H., Waguri S., Taguchi K., Kobayashi A., Ichimura Y., Sou Y.S., Ueno I., Sakamoto A., Tong K.I. (2010). The selective autophagy substrate p62 activates the stress responsive transcription factor Nrf2 through inactivation of Keap1. Nat. Cell Biol..

[150] Levine B., Mizushima N., Virgin H.W. (2011). Autophagy in immunity and inflammation. Nature.

[151] Akinduro O., Sully K., Patel A., Robinson D.J., Chikh A., McPhail G., Braun K.M., Philpott M.P., Harwood C.A., Byrne C. (2016). Constitutive Autophagy and Nucleophagy during Epidermal Differentiation. J. Investig. Dermatol..

[152] Choi Y., Bowman J.W., Jung J.U. (2018). Autophagy during viral infection—A double-edged sword. Nat. Rev. Microbiol..

[153] Ichimura Y., Kumanomidou T., Sou Y.S., Mizushima T., Ezaki J., Ueno T., Kominami E., Yamane T., Tanaka K., Komatsu M. (2008). Structural basis for sorting mechanism of p62 in selective autophagy. J. Biol. Chem..

[154] Ong P.Y., Leung D.Y. (2016). Bacterial and Viral Infections in Atopic Dermatitis: A Comprehensive Review. Clin. Rev. Allergy Immunol..

[155] Dalziel K., Dykes P.J., Marks R. (1984). Inflammation due to intra-cutaneous implantation of stratum corneum. Br. J. Exp. Pathol..

[156] Gahring L.C., Buckley A., Daynes R.A. (1985). Presence of epidermal-derived thymocyte activating factor/interleukin 1 in normal human stratum corneum. J. Clin. Investig..

